# *In vivo* assessment of cancerous tumors using boron doped diamond microelectrode

**DOI:** 10.1038/srep00901

**Published:** 2012-11-29

**Authors:** Stéphane Fierro, Momoko Yoshikawa, Osamu Nagano, Kenji Yoshimi, Hideyuki Saya, Yasuaki Einaga

**Affiliations:** 1 Department of Chemistry, Faculty of Science and Technology, Keio University, 3-14-1 Hiyoshi, Yokohama 223-8522, Japan; 2 Division of Gene Regulation, Institute for Advanced Medical Research, Keio University, 35 Shinanomachi Shinjuku, Tokyo 160-8582, Japan; 3 Department of Neurophysiology, School of Medicine, Juntendo University, 2-1-1 Hongo, Bunkyo-ku, Tokyo 113-8421, Japan; 4 JST, CREST, 5, Sanbancho, Chiyoda-ku, Tokyo 102-0075, Japan

## Abstract

The *in vitro* and *in vivo* electrochemical detection of the reduced form of glutathione (L-γ-glutamyl-L-cysteinyl-glycine, GSH) using boron doped diamond (BDD) microelectrode for potential application in the assessment of cancerous tumors is presented. Accurate calibration curve for the determination of GSH could be obtained by the *in vitro* electrochemical measurements. Additionally, it was shown that it was possible to separate the detection of GSH from the oxidized form of glutathione (GSSG) using chronoamperometry measurements. *In vivo* GSH detection measurements have been performed in human cancer cells inoculated in immunodeficient mice. These measurements have shown that the difference of GSH level between cancerous and normal tissues can be detected. Moreover, GSH detection measurements carried out before and after X-ray irradiation have proved that it is possible to assess *in vivo* the decrease in GSH concentration in the tumor after a specific treatment.

Glutathione is a tripeptide, which is found in high concentrations in many living cells and exists in reduced form (L-γ-glutamyl-L-cysteinyl-glycine, GSH) and oxidized form (disulfide form of GSH, GSSG)^1^,^2^,^3^. It is one of the strongest biological anti-oxidant, which means that under oxidative stress, GSH will be oxidized to GSSG, which in turn will be immediately reduced back to GSH by an enzyme (glutathione reductase)^1^,^2^,^3^,^4^. Because of this fast turnover, GSH concentrations in living cells are usually much higher compared to GSSG. For this reason, the ratio of GSH to GSSG often serves as a sensitive indicator of oxidative stress and is a key marker for the redox status of cells^1^,^2^,^3^,^4^.

Additionally, it was reported that GSH concentrations in cancerous cells are much higher when compared to healthy tissues^5^. It is thus believed that this high concentration of GSH is the principal reason of the high resistance of cancer stem cells against oxidative stress such as radiotherapy or chemotherapy^5^,^6^.

For this reason, the accurate *in vivo* detection of GSH becomes essential for the assessment of biological characteristics of cancer cells. Several techniques exist in order to measure GSH concentration and most of them involve liquid chromatography with different detection methods such as fluorescence or UV^7^,^8^,^9^. However, most of these methods are based on column derivatization followed by fluorimetric detection or on the conversion to their phenyl or pyridine derivatives followed by UV detection^7^,^8^,^9^. Therefore, these methods require expensive equipment and time-consuming procedures in order to measure GSH concentration. Additionally, these techniques are not suited for *in vivo* GSH detection and thus require tissue samples obtained through biopsy, which is an invasive procedure for the patient. An *in vivo* glutathione concentration measurement technique involving labeling with monochlorobimane before further detection using HPLC was reported^10^. However, this method was tested on plants only (*Arabidopsis*) and it was shown that the concentration measured includes both the reduced and oxidized form of glutathione^10^. Another *in vivo* method for GSH detection in human brain by means of double quantum coherence filtering was also reported^11^. This analysis gave satisfactory results for *in vivo* GSH determination but this indirect detection method involves complicate spectra analysis and time consuming calibration procedures^11^.

Electrochemical methods are a viable alternative due to their simplicity, rapidity and excellent sensitivity. Several electrochemical methods for glutathione detection have already been proposed using electrodes such as platinum, gold or gold/mercury^12^,^13^,^14^. These methods have shown to be quite efficient for *in vitro* glutathione determination. However, gold electrodes usually require time consuming pretreatments in order to get reproducible results. Moreover, the use and preparation of gold/mercury tend to complicate the experimental procedure and therefore, these methods become less suited for real analytical applications. Additionally, adsorption and subsequent fouling on these types of electrode materials often occurs during the oxidation of various organic compounds and thus it becomes difficult to use these electrode materials for *in vivo* GSH detection.

The separate electrochemical detection of both the reduced and oxidized form of glutathione has been reported on boron doped diamond (BDD) electrode^15^. This electrode material was selected due to its outstanding properties compared to other conventional electrode materials such as a wide electrochemical potential window, low background current and weak adsorption of polar molecules^16^,^17^. However, only macroelectrodes were used in that study and the detection was performed in BRB buffer (pH2). Therefore, only *in vitro* electrochemical detection of GSH is possible using the method described in^15^.

In order to solve these problems, in this work, the *in vitro* and *in vivo* detection of the reduced form of glutathione (GSH) using BDD microelectrodes together with cyclic voltammetry and chronoamperometry measurements is presented. *In vivo* measurements are possible when using microelectrodes and taking advantage of the exceptional characteristics of boron doped diamond, a new method is proposed for an accurate and rapid detection of GSH in normal and cancerous cells. Several *in vivo* measurements of GSH in tumors and normal tissues are presented and prove that it becomes feasible to perform an *in vivo* assessment of the biological features and malignancy of tumors using this method.

## Results

### *In vitro* experiments

**Figure 1:**
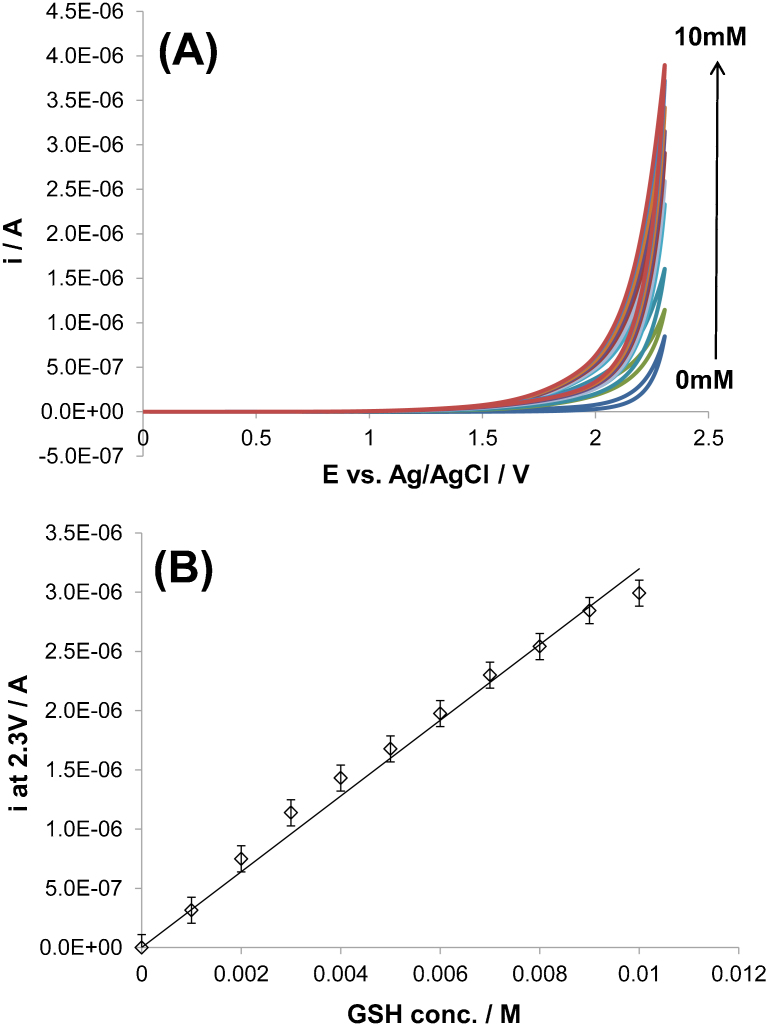
Determination of small concentrations (0–10 mM) of GSH using cyclic voltammetry. (A) Cyclic voltammograms of solutions containing different concentrations of GSH (from 0 to 10 mM) recorded on BDD microelectrode at 0.1 V s^−1^. Potential window between 0 V and 2.3 V vs. Ag/AgCl. (B) Calibration curve for GSH detection: the current reported from (A) at 2.3 V was plotted versus GSH concentration. Support electrolyte: 0.1 M PBS. T = 23°C.

Figure 1A displays cyclic voltammetry measurements recorded at 100 mV s^−1^ on BDD for different concentrations of GSH (ranging from 0 to 10 mM) in 0.1 M PBS. Figure 1A shows that the on-set potential of GSH oxidation to GSSG (reaction 1) is situated approximately at 1.25 V. 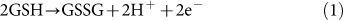
This figure shows also that the oxidation current recorded at a given potential increases with increasing GSH concentration. Using this feature, a calibration curve for GSH determination can be constructed and such curve for a potential of 2.3 V is presented in Figure 1B (the background current measured in 0.1 M PBS in absence of GSH was subtracted from each point and the linear regression was fitted to pass through the origin). This figure shows that accurate calibration (slope: 3.03·10^−4^±0.09·10^−4^, r^2^: 0.988) curve can be obtained for GSH detection in this concentration range. From the data provided on Figure 1B, the lower detection limit using this technique was estimated at 0.3 mM taking three-times the standard deviation.

However, as reported in^18^, the concentration levels of GSH measured in tumorous tissues can reach values around 60 mM. Therefore, the measurements presented in Figure 1 were repeated but for a higher GSH concentration range. Figure S1 displays the calibration curve for GSH detection and for a concentration range between 0 and 80 mM. This figure shows that the precision of such curve obtained from cyclic voltammetry measurements remains acceptable (slope: 3.36·10^−4^±0.06·10^−4^, r^2^: 0.998) for higher GSH concentrations.

It is worthwhile to mention, that successive voltammetric scans performed on BDD for GSH solutions have shown that the activity of the electrode material decreases with successive measurements due probably to the formation of a polymeric film at the surface as already observed with other compounds such as dopamine^16^,^19^. Even for low concentrations of GSH (2 mM), a decrease in the oxidation current recorded at 2.3 V of approximately 8% was observed after 5 successive voltammetric scans. However, the activity of the BDD electrode can be recovered through cathodic treatment. In fact, Figure S2 shows the differences between cyclic voltammograms for 0.1 M PBS solution and recorded: (a) before GSH detection measurements, (b) after 100 voltammetric scans recorded in the presence of 10 mM GSH and (c) after 20 minutes of cathodic treatment at −3 V performed after the GSH detection measurements. This Figure S2 shows clearly that GSH detection measurements induced a decrease of about 80% of the current recorded in PBS. However, the figure shows also that cathodic treatment can recover the activity of the BDD electrode.

In order to perform an accurate assessment of the cancerous tumor, it would be advantageous to be able to distinguish between the concentrations of the reduced form (GSH) and oxidized form (GSSG) of glutathione between healthy and cancerous cells.

**Figure 2:**
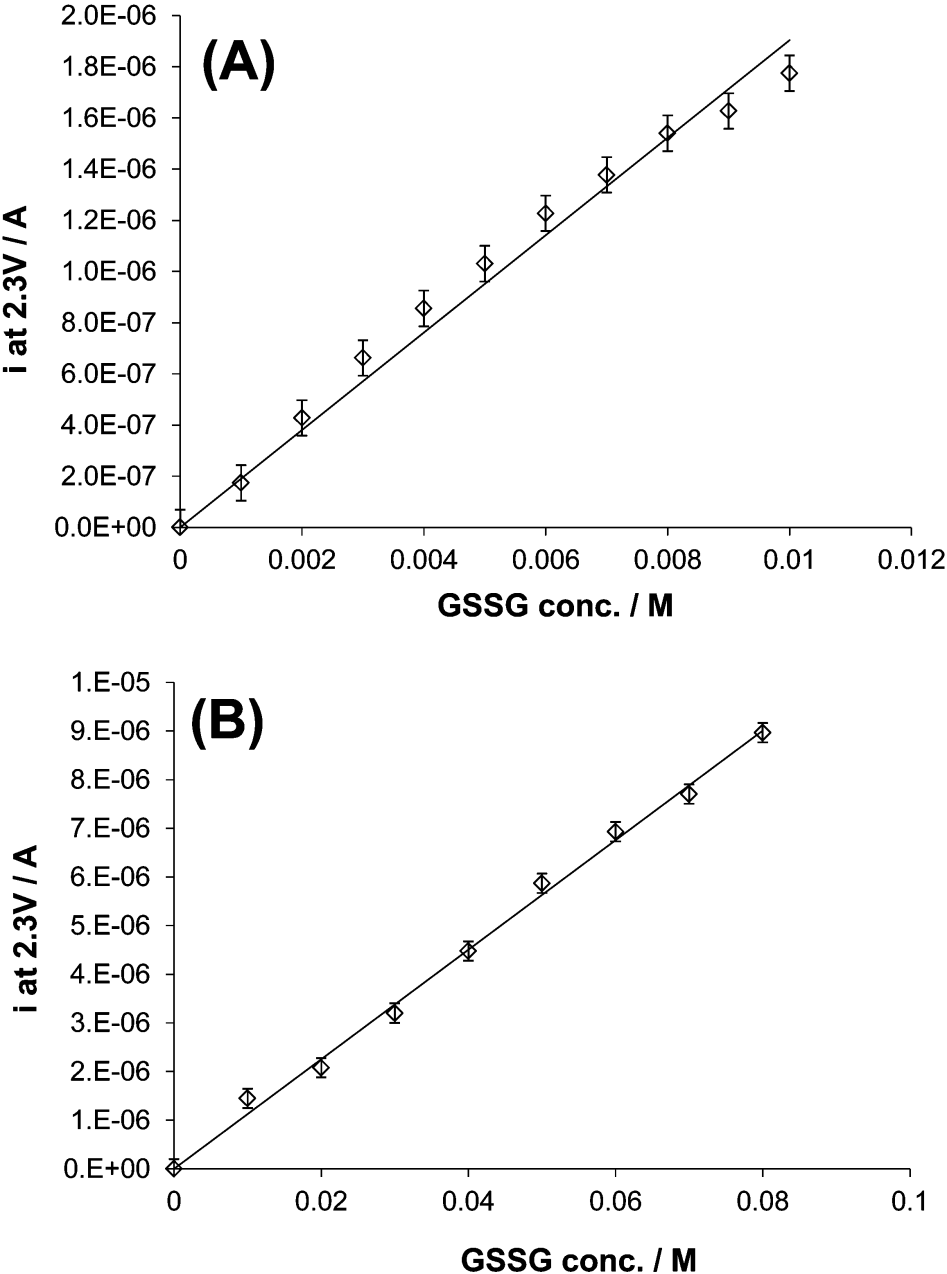
Calibration curve for the determination of GSSG concentration. The currents recorded at 2.3 V vs. Ag/AgCl during cyclic voltammetry measurements performed on BDD microelectrode and using solutions containing different concentrations of GSSG ranging from: (A) 0 and 10 mM and (B) 0 and 80 mM, were plotted versus GSSG concentration. Support electrolyte: 0.1 M PBS. T = 23°C.

However, cyclic voltammetry measurements performed in the presence of GSSG have shown that if the chosen potential value for the calibration plot is 2.3 V, accurate detection of GSSG can be achieved. In fact, Figure 2A and 2B display such calibration curve for low (between 0 and 10 mM; slope: 1.8·10^−4^±0.07·10^−4^, r^2^: 0.988) and high (between 0 and 80 mM; slope: 1.12·10^−4^±0.03·10^−4^, r^2^: 0.996) concentrations of GSSG, respectively. These figures prove that GSSG can also be detected using the same technique previously used for GSH.

**Figure 3:**
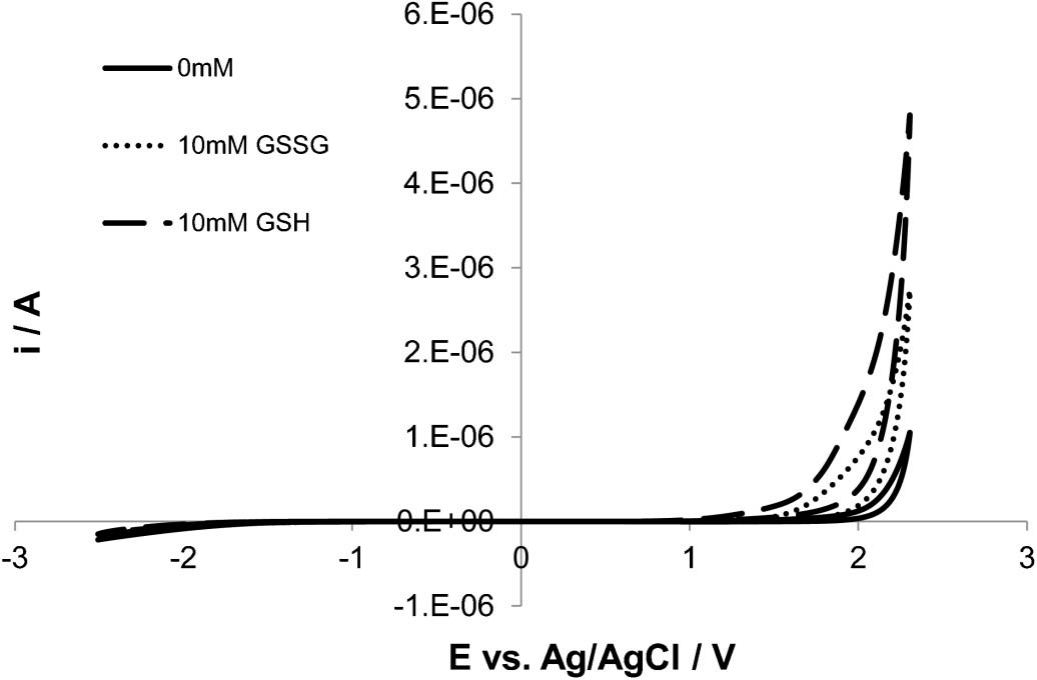
Difference of oxidation potential between GSH and GSSG. Cyclic voltammograms recorded on BDD microelectrode at 0.1 V s^−1^ for two solutions containing 10 mM GSH and 10 mM GSSG, respectively. The background current is also presented for comparison. Potential window between −2.5 V and 2.3 V vs. Ag/AgCl. Support electrolyte: 0.1 M PBS. T = 23°C.

However, the on-set potentials of GSH and GSSG are slightly different as shown in the cyclic voltammogram comparison presented in Figure 3. Therefore, if one carries out the detection using chronoamperometric measurements at carefully chosen potentials (between 1.2 and 1.5 V in this case), it would be possible to detect only GSH and thus separate it from GSSG.

**Figure 4:**
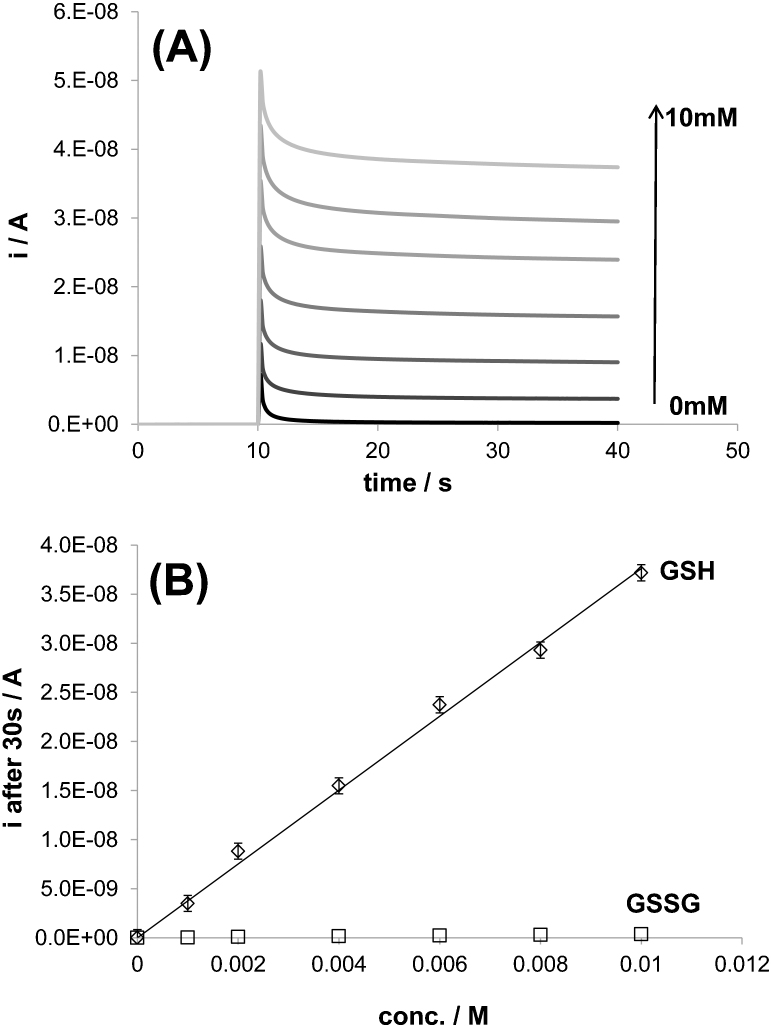
Calibration curve for small concentrations (0–10 mM) of GSH using chronoamperometry. (A) Chronoamperometry measurements recorded on BDD microelectrode and using GSH solutions with different concentrations (from 0 to 0.01 mM). 0 V was applied for 10 seconds before performing a potential step of 1.3 V vs Ag/AgCl. (B) Calibration curve for GSH detection constructed from the measurements presented in (A): the current density measured after 30 s was plotted as a function of GSH concentration. A calibration curve for GSSG was constructed separately under the same conditions and has been added in (B) for a matter of comparison. Support electrolyte: 0.1 M PBS. T = 23°C.

Figure 4A displays chronoamperometry measurements performed at 1.3 V and in 0.1 M PBS solutions containing between 0 and 10 mM GSH. This figure shows clearly that the anodic current recorded increases with increasing GSH concentrations. In fact, it is possible to construct highly accurate calibration plots for GSH detection (slope: 3.69·10^−6^±0.09·10^−6^, r^2^: 0.997) using this technique as shown in Figure 4B. Moreover, in the same figure, it is also proven that the differences in current intensity between 0.1 M PBS in the presence and absence of 10 mM GSSG is almost negligible compared to the plot related to GSH. Therefore, it is possible to separate the detection of the reduced form from the oxidized form of glutathione using this technique.

Moreover, in the case of a solution containing both GSH and GSSH, it would still be possible to separate the detection of GSH from GSSG if the potential applied during the chronoamperometry measurement is carefully selected i.e. before the on-set potential of GSSG oxidation. However, it would not be possible to separate the detection of GSSG from GSH. In fact, if the on-set potential of GSSG oxidation was selected for the chronoamperometry measurement, GSH oxidation would also occur and the current measured would be related to the oxidation of both GSH and GSSG.

**Figure 5:**
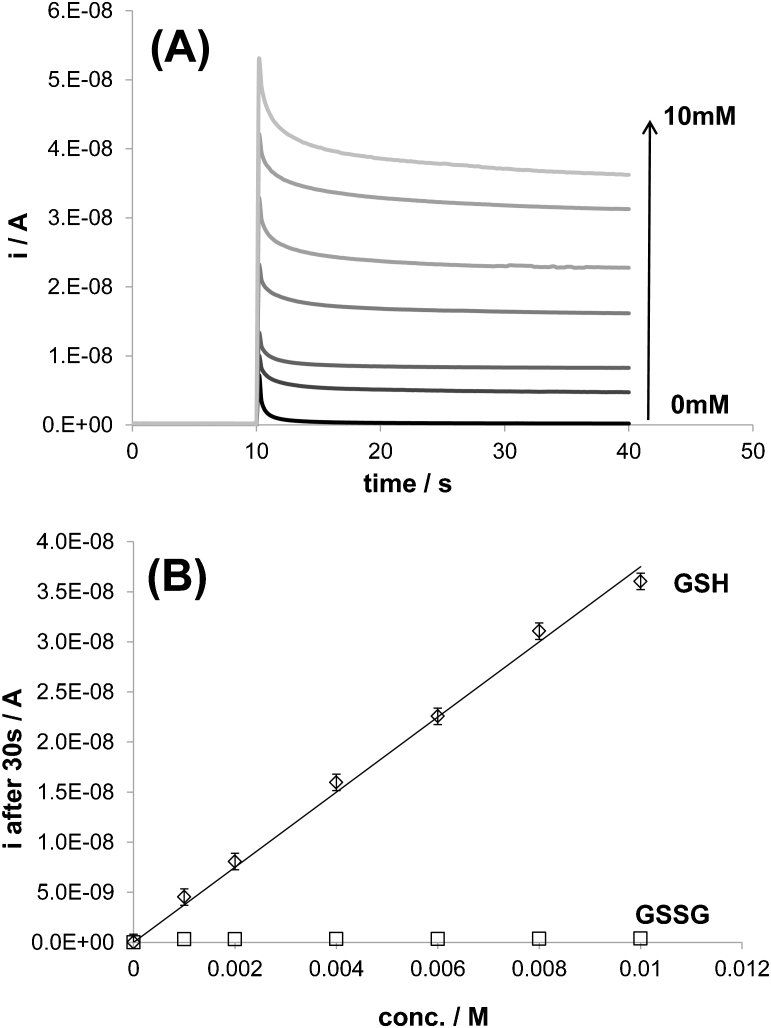
Calibration curve for small concentrations (0–10 mM) of GSH using chronoamperometry at body temperature (37°C). (A) Chronoamperometry measurements recorded on BDD microelectrode and using GSH solutions with different concentrations (from 0 to 0.01 mM). 0 V was applied for 10 seconds before performing a potential step of 1.3 V vs Ag/AgCl. (B) Calibration curve for GSH detection constructed from the measurements presented in (A): the current density measured after 30 s was plotted as a function of GSH concentration. A calibration curve for GSSG was constructed separately under the same conditions and has been added in (B) for a matter of comparison. Support electrolyte: 0.1 M PBS. T = 37°C.

In order to simulate *in vivo* experiments, the chronoamperometry measurements and corresponding calibration curves presented in Figure 4A and 4B have been repeated but at 37°C. The results are displayed in Figure 5A and 5B and they show that an increase in temperature from 23 to 37°C has no significant influence on GSH detection using this method.

It is worthwhile to notice that the construction of the calibration curves presented as well as the separation of the detections of GSH and GSSG was possible due to the unique properties of boron doped diamond (large potential window and low capacitive current). In fact, it was reported in^15^ that the stability and accuracy of GSH and GSSG detection on other electrode materials such as glassy carbon were far inferior when compared with BDD due to the high background current recorded on glassy carbon.

### *In vivo* experiments

**Figure 6:**
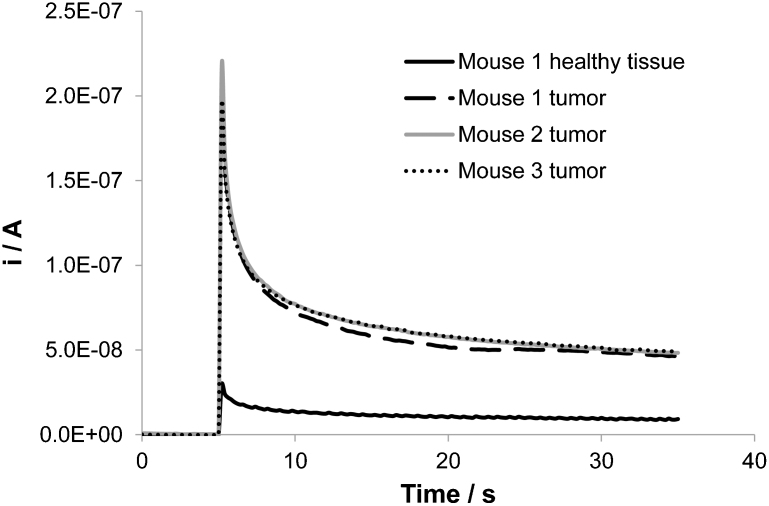
Assessment of the biological features of HSC-2-derived xenograft tumor: difference between normal and tumorous tissue. Chronoamperometry measurements recorded on BDD microelectrode at 1.3 V vs. Ag/AgCl: in normal tissue (flank) of mouse 1, in the HSC-2-derived xenograft tumor of mouse 1, in the HSC-2-derived xenograft tumor of mouse 2 and in the HSC-2-derived xenograft tumor of mouse 3. All tumors had been inoculated for two weeks. T = 37°C.

Figure 6 shows chronoamperometry measurements recorded at 1.3 V in subcutaneous normal and tumor tissues of nude mice. Measurements were performed in normal tissue and inside xenograft tumors derived from human squamous cell carcinoma cells (HSC-2 cell) that had been inoculated in three different mice for two weeks. This figure shows that the anodic current recorded increases when the measurement is performed inside the tumor when compared to the healthy tissue. Moreover, measurements performed in three distinct individuals but under the same conditions give similar results thus proving that the method is highly reproducible. If one considers that the difference between the different currents recorded is almost exclusively due to the difference in GSH concentration inside the tissue (due to its affinity for oxidation), this measurement proves that it is possible to perform an *in vivo* assessment of the tumor using this method.

It is worthwhile to notice though that biofouling of the BDD electrode occurs after one measurement, which induces a decrease in the current difference between the measurements performed in normal and tumorous tissues. For this reason, it is necessary to perform a cathodic treatment (−3 V for 20 minutes) in 0.1 M PBS between each measurement. Additionally, if one wishes to obtain reliable results using this method, the *in vivo* current measurement should not last more than 5 seconds.

In order to attest if a decrease in GSH concentration inside the tumor can be detected, among the three mice used for our investigation; two mice were X-ray irradiated for two minutes but at different levels (2 Gy and 6 Gy). X-ray irradiation has shown to decrease considerably the amount of GSH in living tissues as reported in^20^.

**Figure 7:**
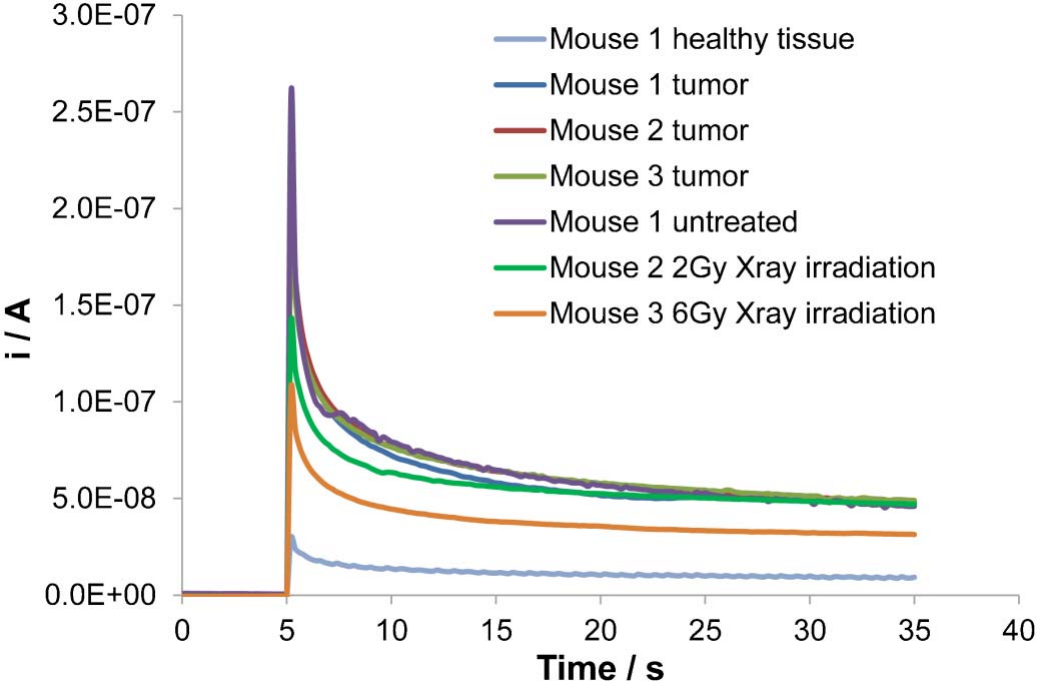
Assessment of the biological features of HSC-2-derived xenograft tumor before and after X-ray treatment. The results presented in figure 5 are compared with chronoamperometry measurements recorded on BDD microelectrode at 1.3 V vs. Ag/AgCl: in the HSC-2-derived xenograft tumor of mouse 1 (untreated; measurement performed three hours after the results presented in Figure 5), in the HSC-2-derived xenograft tumor of mouse 2 (three hours after the tumor had been X-ray irradiated for 2 minutes at 2 Gy) and in the HSC-2-derived xenograft tumor of mouse 3 (three hours after the tumor had been X-ray irradiated for 2 minutes at 6 Gy). T = 37°C.

Then, after three hours, GSH detection measurements were carried out in the HSC-2-derived xenograft tumors of the three mice (among which only two were X-ray irradiated) and compared with the results presented in Figure 6. Figure 7 displays this comparison and it shows that the current density recorded in the tumor decreased significantly after irradiation whereas the current density measured in the tumor remained unchanged for the mouse, which was not irradiated. Moreover, the decrease in current density increased with increasing intensity of the irradiation. This figure thus shows that it is possible using this technique to detect the variation of GSH concentrations in cancerous tumors before and after a specific treatment.

## Discussion

In the present work, *in vitro* and *in vivo* detection of the reduced form of glutathione was performed on BDD microelectrodes using cyclic voltammetry and amperometry measurements for potential application in cancerous tumors assessment.

In summary, first, it is possible to build highly accurate calibration curves for the reduced form of glutathione (GSH) detection using BDD microelectrode and in 0.1 M PBS solution (pH7.4) using cyclic voltammetry and chronoamperometry measurements. Second, the separate detection of the reduced and oxidized forms of glutathione (GSSG) is difficult to achieve using cyclic voltammetry measurements due to the similar oxidation on-set potential of oxidation. However, when using chronoamperometry measurements at a carefully selected potential (1.3 V vs Ag/AgCl), it is possible to measure only the concentration of the reduced form as its oxidation starts slightly before GSSG. Finally, GSH detection measurements carried out in subcutaneous xenograft tumors derived from human cancer cells in immunodeficient mice have shown that it is possible to measure *in vivo* the difference in concentration of GSH between cancerous and healthy tissues with a high reproducibility. Moreover, measurements performed before and after X-ray irradiation have shown that the variation of GSH concentration inside the tumor could be detected.

## Methods

### Chemicals and materials

Glutathione (GSH), glutathione disulfide (GSSG), disodium hydrogen phosphate dodecahydrate and disodium hydrogen phosphate dihydrate were purchased from Wako. Chemicals were used without further purification.

### Preparation of BDD microelectrodes

**Figure 8:**
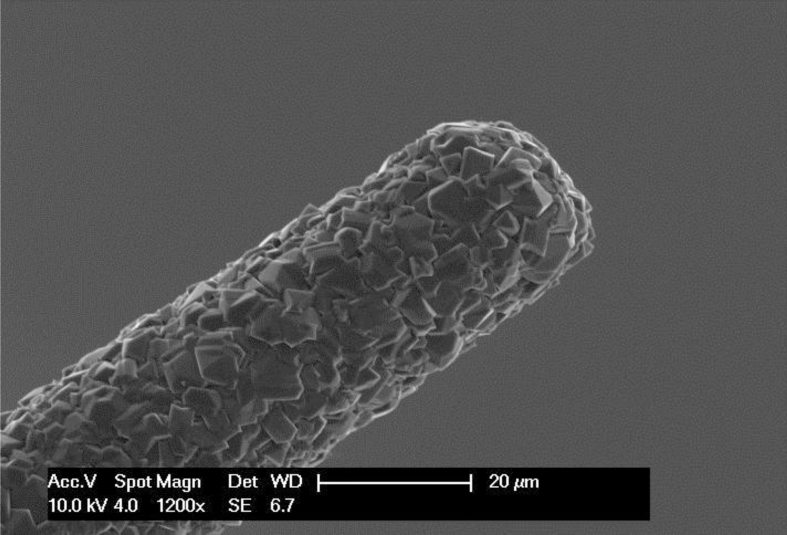
SEM image of the BDD microelectrode. The image shows the tip of the needle shaped BDD microelectrode.

BDD microelectrodes were prepared using a microwave plasma-assisted chemical vapor deposition (MPCVD) set-up (ASTeX Corp.). Acetone was used as a carbon source, and B(OCH_3_)_3_ as a source of boron. The concentration of the latter was 0.1% w/w in the source. The surface morphology and crystalline structures were characterized using scanning electron microscopy (SEM). Figure 8 displays a SEM image of the tip of the BDD microelectrode. BDD was deposited on a tungsten needle (20μm in diameter) in an MPCVD chamber at 2.5 kW using high-purity hydrogen as a carrier gas. A portion of the needle was isolated using a glass capillary in order to define the working surface area so that about 1 mm remains uncovered by the capillary. The film quality was confirmed by Raman spectroscopy (not shown). The BDD microelectrodes were pre-treated by ultrasonication in 2-propanol for about 10 minutes followed by rinsing with high-purity water to remove any organic impurities that may have remained within the BDD film after deposition in the MPCVD chamber.

### Electrochemical measurements

*In vitro* electrochemical measurements were carried out in a single-compartment cell using an AUTOLAB PGSTAT potentiostat at room temperature (23°C). The reference electrode was Ag/AgCl, the counter electrode was a platinum wire and the working electrode was the BDD microelectrode. Considering the tungsten needle as a cylinder, the working geometric area was about 6.3·10^−4^ cm^2^. The electrochemical detection of glutathione was performed in 0.1 M phosphate buffer saline solution (PBS), which is prepared by mixing disodium hydrogen phosphate dodecahydrate and dehydrate until reaching a pH of 7.4. For the in vivo electrochemical experiments, the reference electrode was Ag/AgCl, the counter electrode was a silver wire and the working electrode was the BDD microelectrode. All potentials quoted in this work are with respect to the Ag/AgCl reference electrode (0.2 V vs. SHE).

### Mouse xenograft tumor model

Human oral squamous cell carcinoma cell line HSC-2 (1·10^6^) were implanted subcutaneously in the flank of nude mice. Two weeks after transplantation, the GSH concentration in the tumor tissues was measured using a BDD microelectrode. In order to measure *in vivo* the concentration of GSH, the BDD needle, the silver wire and the Ag/AgCl wire were inserted in the tissue to be analyzed to a depth between 2 and 3 mm. All animal experiments were performed in accordance with protocols approved by the Ethics Committee of Keio University.

## Author Contributions

Y.E., H.S. and S.F. conceived the project and designed the experiments. M.Y. and S.F. performed the experiments. K.Y. helped for the *in vivo* experiments. O.N. and S.F. analyzed the data. S.F. wrote the manuscript. All authors reviewed the manuscript.

## Supplementary Material

Supplementary Information Supporting information
