# Supplementary material for: In vivo assessment of cancerous tumors using boron doped diamond microelectrode
Source: Sci Rep. 2012 Nov 29;2:901. doi: 10.1038/srep00901 (PMC3509351; doi:10.1038/srep00901)
Supplement: Supplementary Information — Supporting information [file srep00901-s1.doc]

**Supporting information:**

***In vivo* assessment of cancerous tumors using boron doped diamond microelectrode**

Stéphane Fierro*^1^, Momoko Yoshikawa^2^, Osamu Nagano^2^, Kenji Yoshimi^3^,

Hideyuki Saya^2,4^ and Yasuaki Einaga*^1,4^

^1^ Department of Chemistry, Faculty of Science and Technology, Keio University, 3-14-1 Hiyoshi, Yokohama 223-8522, Japan

^2^ Division of Gene Regulation, Institute for Advanced Medical Research, Keio University, 35 Shinanomachi Shinjuku, Tokyo 160-8582, Japan

^3^ Department of Neurophysiology, School of Medicine, Juntendo University, 2-1-1 Hongo, Bunkyo-ku, Tokyo 113-8421, Japan

^4^ JST, CREST, 5, Sanbancho, Chiyoda-ku, Tokyo 102-0075, Japan

*: corresponding authors

E-mails: sfierro@chem.keio.ac.jp and einaga@chem.keio.ac.jp


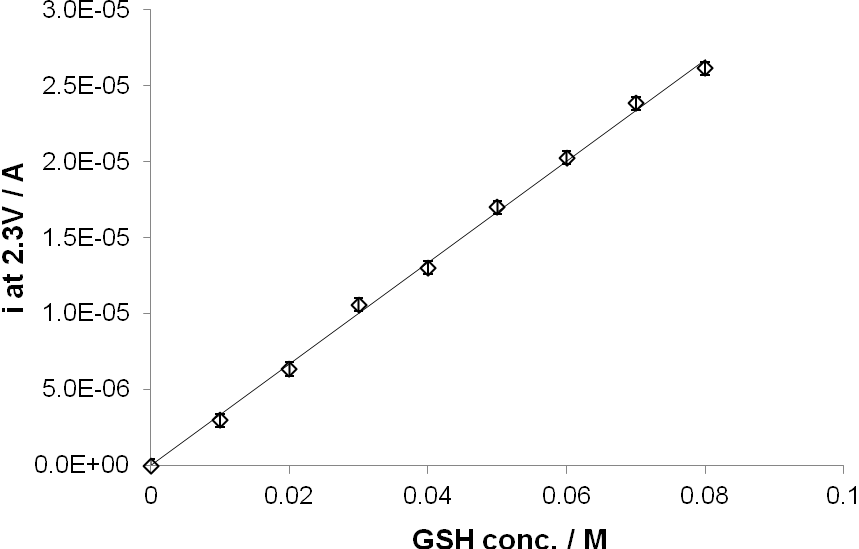


**Figure S1**:

Calibration curve for high concentrations (0-80mM) of GSH using cyclic voltammetry. The currents recorded at 2.3V vs. Ag/AgCl during cyclic voltammetry measurements performed on BDD microelectrode and using solutions containing different concentrations of GSH ranging from 0 and 80mM were plotted versus GSH concentration. Support electrolyte: 0.1M PBS. T=23ºC.


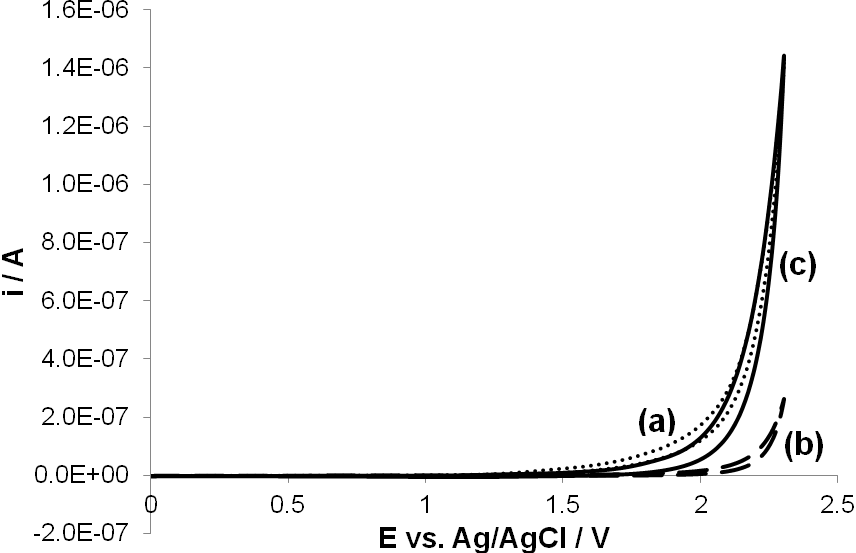


**Figure S2**:

Recovery of the activity of the BDD electrode from biofouling. Cyclic voltammograms for 0.1M PBS solution and recorded on BDD microelectrode: (a) before GSH detection measurements, (b) after 100 voltammetric scans recorded in the presence of 10mM GSH and (c) after 20 minutes of cathodic treatment at -3V performed after the GSH detection measurements. Potential window between 0 V and 2.3 V vs. Ag/AgCl. Scan rate: 0.1V s^-1^. Support electrolyte: 0.1M PBS. T=23ºC.
